# The Genus *Diospyros*: A Review of Novel Insights into the Biological Activity and Species of Mozambican Flora

**DOI:** 10.3390/plants12152833

**Published:** 2023-07-31

**Authors:** Adriana Ribeiro, Rita Serrano, Isabel B. Moreira da Silva, Elsa T. Gomes, João F. Pinto, Olga Silva

**Affiliations:** Research Institute for Medicines (iMed.ULisboa), Faculty of Pharmacy, Universidade de Lisboa, 1649-003 Lisboa, Portugal; ribeiroadriana@edu.ulisboa.pt (A.R.); rserrano@edu.ulisboa.pt (R.S.); isabelsilva@edu.ulisboa.pt (I.B.M.d.S.); eteixeiragomes@sapo.pt (E.T.G.); jfpinto@ff.ul.pt (J.F.P.)

**Keywords:** antimicrobial activity, anti-inflammatory activity, cytotoxicity, *Diospyros*, ethnomedicinal practice, herbal medicine, infectious diseases

## Abstract

Species of the *Diospyros* L. genus (*Ebenaceae* family) have been largely used in traditional medicine for the treatment of several diseases, especially infectious ones. To date, active major compounds such as naphthoquinones, triterpenoids, and tannins have been isolated and pharmacologically validated from *Diospyros* species. The present study summarizes the information available in the literature on the species described in the Flora of Mozambique. To do so, scientific databases (e.g., PubMed, Scopus, Web of Science, and Google Scholar) were searched using various keywords and Boolean connectors to gather and summarize the information. Of the 31 native and naturalized species in the Flora of Mozambique, 17 are used in different regions of Africa and were described for their traditional uses. They were reported to treat more than 20 diseases, mostly infectious, in the gastrointestinal and oral cavity compartments. This work provides an overview of the therapeutical potential of *Diospyros* species and explores novel insights on the antimicrobial potential of extracts and/or isolated compounds of these Mozambican species.

## 1. Introduction

The genus *Diospyros* L. (*Ebenaceae* family) contains species that have been recognized and used in traditional medicine (extended ethnomedical use) and have potential new health benefits supported by in vitro biological, in vivo pharmacological, and clinical tests [1,2,3,4]. Furthermore, within certain cultures or communities, various traditional systems have used all plant parts of this botanical genus (leaf, fruit, bark, twig, hardwood, and root) as herbal medicines [1,4]. 

Beyond their pharmacological value, *Diospyros* spp. have distinct and complementary important qualities, namely valuable wood, and edible fruits, which provide significant economic benefits and are recognized and utilized in various industrial and commercial sectors [1,4]. 

Generally, *Diospyros* spp. are tree shrubs or subshrubs with entire alternate leaves, solitary flowers, and fleshy fruits (berries) with usually two or more seeds. The characteristics of the leaves and flowers of these species are often used to identify fossil casts [5,6,7].

*Diospyros* species are predominantly distributed between the tropics, and the most notable diversity of this botanical genus occurs in Africa [5,6,8]. As confirmed in The Plant List [9], the WFO Plant List currently contains 1575 species related to the genus *Diospyros*, of which 734 have accepted scientific names [10]. Regarding the Mozambican flora, the genus is represented by 31 species (Table 1), corresponding to 18 accepted scientific name species, seven accepted subspecies (subsp.), three species that are considered synonyms, and three species that are not yet in the WFO plant list as of 12 February 2022 [10,11,12].

Concerning primary health care (PHC), herbal medicines are used by 80% of the African population, and more than 70% of the population of Mozambique uses such medicines for treating all diseases [13,14,15]. For instance, several *Diospyros* species with antimicrobial potential have been reported [4,16,17,18]. Worldwide, the magnitude of infectious diseases (ID), encompassing antimicrobial resistance (AMR), represents a major health problem (approximately 700,000 people die every year) [19,20]. Infectious diseases have a high impact in Africa, particularly in Mozambique [21].

Most of the native and naturalized *Diospyros* species of Mozambique’s flora are generally recognized as traditionally used in different regions of Africa to treat different diseases, with a particular focus on infections affecting the gastrointestinal tract and oral cavity. This work will present a comprehensive overview of the therapeutic potential of Mozambican *Diospyros* species based on chemical, biological, and toxicological experimental data, particularly addressing its antimicrobial properties and including comparative elements concerning the biological activity of other *Diospyros* species.

## 2. Results

### 2.1. Ethnomedical Use of Diospyros Species of Mozambican Flora

Table 2 shows the results of the collected ethnomedical data from seventeen Mozambican species, namely *D. abyssinica*, *D. anitae*, *D. ferrea*, *D. kabuyeana*, *D. loureiriana* subsp. *loureiriana*, *D. lycioides* subsp. *sericea*, *D. mafiensis*, *D. mespiliformis*, *D. rotundifolia*, *D. mafiensis*, *D. mespiliformis*, *D. quiloensis*, *D. rotundifolia*, *D. squarrosa*, *D. usambarensis*, *D. verrucosa*, *D. villosa*, *D. villosa* var. *parvifolia*, *D. whyteana*, and *D. zombensis*. In addition, information is given on the part of the plant used as medicine, the manufacturing process of the traditional formulation, the main traditional therapeutic use, and the country from which the information originates.

**Figure 1:**
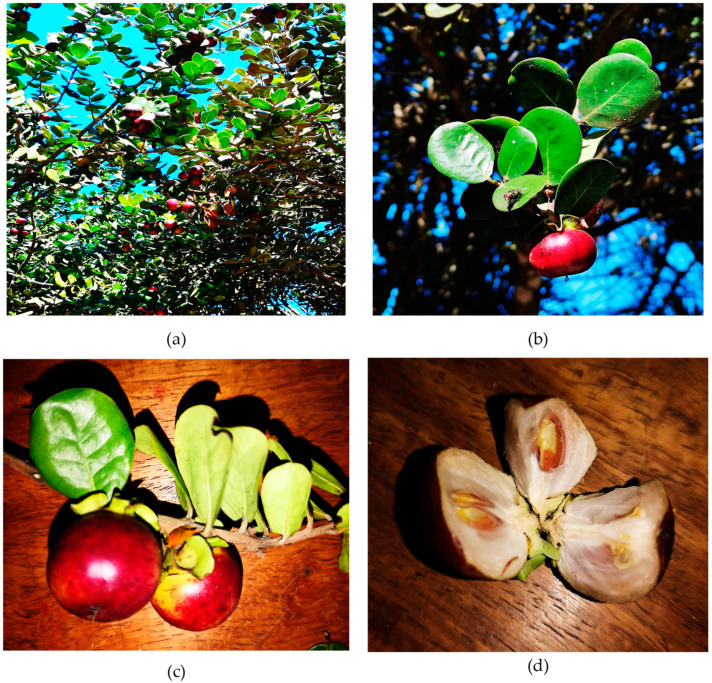
*Diospyros rotundifolia*: (**a**) aspect in its natural habitat; (**b**,**c**) details of leaf and fruit; (**d**) transverse view of the fruit with the seeds. Photography by Elsa Gomes.

The results show that 54.8% of the total *Diospyros* species from Mozambique are referred to for their traditional use (Table 2). Among these, *D. rotundifolia* (Figure 1), traditionally used to treat diarrhea [22], is a prevailing species of dense undergrowth in the coastal area of the Marracuene District [23].

**Figure 2:**
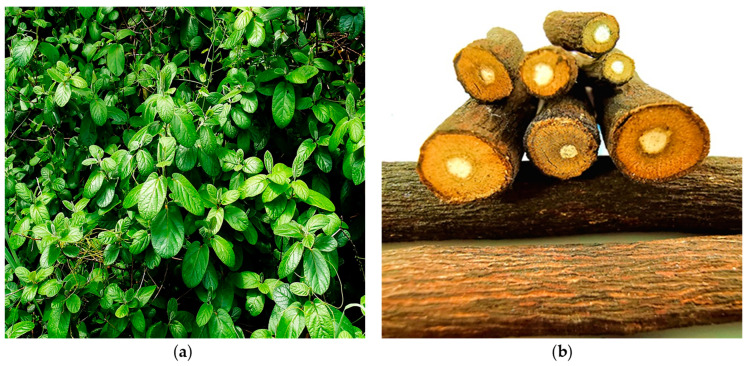
*Diospyros villosa*: (**a**) Aspect in the natural habitat; (**b**) cross-section of the root. Photography by Elsa Gomes (**a**) and Adriana Ribeiro (**b**).

Furthermore, among the *Diospyros* species present in the Mozambican flora, *D. villosa* (Figure 2) is a species with a well-established traditional use of both leaf [24] and root [25]; the latter mainly used as a toothbrush for hygiene purposes [26].

*Diospyros* species have been reported to be used to treat the signals and symptoms of over 20 diseases. Two of these species (*D. abyssinica* and *D. mespiliformis*) have been mentioned most frequently and are used in two to five different countries in Africa (Table 2).

Based on the diverse description in the literature for the human use of the different parts of *Diospyros*, the results are grouped into infectious diseases (antibacterial, antifungal, anthelminthic, and antiviral); gastrointestinal (diarrhea, dysentery, emetic, flatulence, and other gastrointestinal disorders), oral cavity (oral hygiene, healing of oral wounds, and toothaches); urogenital (anti-hemorrhagic, dysmenorrhea, and infertility); skin diseases (dermatitis, fresh wounds, bedsores, and rashes); musculoskeletal (body pain, bruises, painful fractures, and rheumatism); and others conditions (diabetes, internal injuries, antidotes, hemostatic agents, and snake bites).

**Figure 3:**
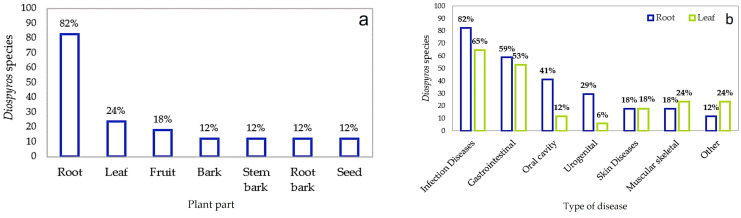
Traditional use of *Diospyros* species: (**a**) plant part used; (**b**) type of disease.

Among all the different *Diospyros* plant parts used in traditional medicine (Figure 3), the root is the most-used part (82%, Figure 3a) and is most used to treat infectious diseases. In the treatment of gastrointestinal disorders, it corresponds to 59%, for oral cavity infections, 41%, and for skin diseases, 18%, as well as for the management of other conditions, comprising 12% (Figure 3b).

The leaf is the second-most used part of the *Diospyros* species, but it is used in a similar percentage (18%) to the root to treat skin conditions and more commonly (24%) for musculoskeletal bruises, painful fractures, body aches, and rheumatism (Figure 3a,b).

**Table 2:** Reported ethnomedical use of Mozambican *Diospyros* species.

Species	Part Used	Preparation Method	Traditional Use	Country	Ref.
*D. abyssinica*					
	leaf	decoction	malaria	Mali	[4,27]
			wound healing		
	fruit (dry)	decoction	astringent and cholagogue		
			gastrointestinal disorders		
	bark	unspecified	astringent and antipyretic		
	root	decoction	antihelminthic		
			abdominal pain, dysentery, and diarrhea		
	leaf	juice	snake bite	Mali, Guinea Zimbabwe	[28]
	bark		astringent		
	bark	decoction	internal injuries	Kenya	[29]
			laxative		
			rash		
			malaria and ringworm		
	leaf	squeeze and apply	ringworm	Uganda	[30]
	seed		wound healing		
	leaf	juice	tropical ulcer (skin and soft tissue polymicrobial infection, feet, or lower legs localized)		[31]
	tuber	decoction	upset stomach		[32]
*D. anitae*					
	root	unspecified	dental hygiene	Mozambique	[33]
			healing of oral wounds		
*D. ferrea*					
	fruit	unspecified	diarrhea and sore throats	India	[34]
			internal bleeding		
			renal lithiasis		
	root	unspecified	anti-hemorrhagic		[35,36]
			infertility		
	bark		oral hygiene		
			skin diseases		
*D. kabuyeana*					
	root	unspecified	antiviral	Tanzania	[37]
*D. loureiroana* subsp. *loureiroana*					
	root	chewing stick	oral hygiene	South Africa East Africa	[4]
*D. lycioides* subsp. *sericea*					
	root bark	decoction	bloody feces	South Africa South Central Zimbabwe	[38,39,40]
			dysentery		
			headache		
	root	chewing stick	infertility	Namibia Zambia	[41]
*D. mafiensis*					
	root	unspecified	diarrhea	Mozambique Tanzania	[42]
			leprosy		
			skin diseases (including fungal infections)		
*D. mespiliformis*					
	leaf	decoction	analgesic and antipyretic	Central Southern Eastern Western Africa	[27,43,44,45,46,47]
			antihelminthic		
			dermatomycosis		
			fungal infections		
			induction of childbirth		
			hemostatic agent		
			malaria, pneumonia, and trypanosomiasis		
			sexually transmitted diseases		
	leaf and bark	decoction	diarrhea and dysentery		
			leprosy		
			oral infections		
			whooping cough		
	leaf	decoction	bruises, bedsores, rash, and wounds		
			ringworm		
	root	chewing stick	oral hygiene		
	leaf, bark and root	decoction	toothache	Burkina Faso	[48]
	leaf	decoction	antipyretic	Ghana	[27]
			dermatitis		
			diarrhea and dysentery		
			malaria		
	fruit	decoction	headache		
			pneumonia		
			rheumatism		
	stem bark	decoction	malaria and pneumonia		
	root	decoction	infection with fever		
	leaf	decoction	antipyretic	Nigeria	[49,50,51]
			antidote for a variety of poisonous substances		
			diarrhea and dysentery		
			haemostatic agent		
			oral infections		
			wound healing		
	root	decoction	malaria and oral candida infection (used as mouthwash, management of HIV/AIDS opportunistic diseases)	Zambia	[44,52]
	root	infusion	abdominal pain, body and heart pain	South Central Zimbabwe	[53]
	seed	unspecified	antibacterial	Guinea	[4]
*D. quiloensis*					
	stem bark	decoction	malaria	Zambia	[44]
			sexually transmitted diseases		
*D. rotundifolia*					
	root	not report	diarrhea	South African	[22]
*D. squarrosa*					
	root	not report	sexually transmitted diseases	Tanzania	[37]
*D. usambarensis*					
	root bark	unspecified	schistosomiasis	Malawi	[54]
	root	chewing stick	oral hygiene	Tanzania	[55,56]
		decoction	fungal infections and overt symptoms of type 2 diabetes (i.e., polyuria, polydipsia, excessive thirst, and sweating)		
*D. verrucosa*					
	root	unspecified	leprosy	Tanzania	[4,57]
*D. villosa*					
	leaf	unspecified	gastrointestinal disorders	South African	[24]
			painful fractures		
	root	decoction	gastrointestinal disorders	Mozambique	[4,25,58]
			laxative		
			musculoskeletal system		
		toothbrush	oral hygiene		
		decoction	wounds (skin/subcutaneous tissue)		
*D. villosa* var. *parvifolia*					
	leaf	infusion	emetic	South Africa	[59]
	root		antihelminthic emetic and flatulence gastrointestinal disorders		
*D. whyteana*					
	root	unspecified	antibacterial	South Africa	[60]
			dysmenorrhea		
			rash		
*D. zombensis*					
	root bark	unspecified	schistosomiasis	Malawi	[4,61]

The majority of documented medicinal uses of *Diospyros* species are attributed to their effectiveness in treating microbial infections, encompassing bacterial, fungal, and parasitic infections. These include conditions such as diarrhea, dysentery, and various skin and oral cavity infections.

### 2.2. Chemical Composition of Mozambican Diospyros Species

The main classes of chemical constituents identified in *Diospyros* species from the Mozambican flora are listed in Table 3.

The presence of phenolic acid derivatives, like flavonoids and naphthoquinones (NQs), particularly 1,4-naphthoquinones (1,4-NQs), and terpenoids, mainly triterpenoids (especially lupan, ursane, oleanane derivatives) [3,4,17,62,63] and tetraterpenoids (carotenoids), have been reported [4]. Other chemical constituents in these *Diospyros* species include hydrocarbons, lipids, amino acids, and sugars [1,4,5,62].

**Table 3:** Chemical compounds identified in Mozambican *Diospyros* species.

Species	Part Used	Chemical Class	Compounds	Extract	Ref.
*D. abyssinica*					
	root bark	naphthoquinone	plumbagin (2-methyl-5-hydroxy-1,4-naphthoquinone)	P. ether, CF, DCM, H_2_O, MeOH, EtOH 80%	[28]
	stem bark	naphthoquinone	diospyrin, isodiospyrin		[64,65]
	leaf	triterpenoid	betulinic acid, betulin and lupeol	MeOH	
*D. consolatae*					
	n.r	triterpenoid	betulinic acid, betulin and lupeol	n.r	[4]
	n.r	naphthoquinone	diosindigo A	n.r	[4]
*D. dichrophylla*					
	seed	naphthoquinone	isodiospyrin	Hex	[66]
*D. ferrea*					
	leaf	triterpenoid	pregnenolone and androstan-6-one	MeOH	[67]
	n.r		*β*-sitosterol	n.r	[4]
	leaf	monoterpenoid	citronellol	MeOH	[67]
	leaf	diterpenoid	phytol	MeOH	[67]
			thunbergol	EtOAc	
	leaf	triterpenoid	betulin, *α*-amyrin, friedelan-3-one and olen-12-ene	EtOAc	[67]
	fruit	triterpenoid	friedelin, epifriedelinol, lupeol, lupenone, and betulin	n-Hex	[68]
	fruit	triterpenoid	*β*-sitosterol and stigmasterol	n-Hex	[68]
	root fruit	naphthoquinone	7-methyljuglone, isodiospyrin, diosindigo A and 8-hydroxyisodiospyrin	CF, n-Hex	[68,69]
	root	phenol	gallic acid	EtOH	[70]
	leaf	triterpenoid	friedelin, friedelin-3-ol, taraxerol and taraxerone	EtOH	[4]
	n.r	triterpenoid	ursolic acid	n.r	[4]
*D. inhacaensis*					
	stem	naphthoquinone	7-methyljuglone and diospyrin	n.r	[71]
*D. kirkii*					
	n.r	triterpenoid	bauerenol, betulin and lupeol	n.r	[4]
	n.r		*β-*sitosterol	n.r	[4]
	n.r	naphthoquinone	diosindigo A	n.r	[4]
*D. lycioides*					
	branche	naphthalene	Diospyroside A, B, C and D	MeOH	[72]
		naphthoquinone	7-methyljuglone and juglone	MeOH	[41]
		triterpenoid	lupeol and ursolic acid	n.r	[53]
	fruit	naphthoquinone	isodiospyrin and bisisodiospyrin	n.r	[71]
	root, stem	naphthoquinone	7-methyljuglone and isodiospyrin	CF	[71]
	n.r	naphthoquinone	mamegakinone, methylnaphthazarin and 8-hydroxyisodiospyrin	n.r	[4]
*D. mafiensis*					
	root bark	naphthoquinone	diosquinone, diosindigo A, 7-methyljuglone, 3-hydroxiquinone, and 6,8-bisdiosquinone	CF, DCM, MeOH	[42,73,74]
	stem bark	naphthoquinone	7-methyljuglone and diosindigo A		[73]
	leaf	triterpenoid	*α*-amyrin, lupeol and betulinic acid	CF, MeOH	[75]
	bark	naphthoquinone	diosquinone, isodiospyrin, and plumbagin	Ee	[4,52]
	stem bark	triterpenoid	lupeol, betulin, betulinic acid, *α*-amyrin, and bauerenol	CF	[4,76]
*D. mespiliformis*					
	stem bark, leaf, bark	triterpenoid	betulinic acid, betulin, lupeol, bauerenol, and *α*-amyrin	CF, MeOH	[4,76]
	leaf	flavonoid	7-*O*-(4″′-*O-*acetyl)-allopyranosyl(1″′ → 2″)- *β*-glucopyranoside, along with eight flavonoid metabolites—luteolin 3′,4′,6,8-tetramethyl ether, luteolin 4′-*O*-*β*-neohesperidoside, luteolin 7-*O*-*β*-glucoside, luteolin, quercetin, quercetin 3-*O*-*β*-glucoside, quercetin 3-*O*-*α*-rhamnoside, and rutin	n.r	[77]
	root	naphthoquinone	diosquinone, and plumbagin	P. ether	[78]
	root, bark	naphthoquinone	diospyrin	MeOH	[79]
	fruit	naphthoquinone	plumbagin	MeOH	[79]
*D. natalensis*					
	root, stem	naphthoquinone	7-methyljuglone, and diospyrin	n.r	[4]
	n.r	triterpenoid	betulinic acid, *α*-amyrin, and lupeol	n.r	[4]
	n.r	fatty acid	heptacosanoic acid	n.r	[4]
*D. quiloensis*					
	n.r	naphthalene	4,5,6,8-tetramethoxy naphthaldhyde, 5-hydroxy-4,6,8-trimethoxy naphthaldehyde, 4,5,6-trimethoxynaphthalehyde, 4,5-dimethoxynaphthaldehyde, and 5-hydroxy-4-methoxy-2-naphthaldehyde	MeOH	[4]
*D. rotundifolia*					
	n.r	triterpenoid	betulin and lupeol	n.r	[4]
	root	naphthoquinone	7-methyljuglone, neodiospyrin and rotundiquinone	n.r	[71]
	stem	naphthoquinone	7-methyljuglone and diospyrin	n.r	[71]
*D. squarrosa*					
	n.r	naphthoquinone	7-methyljuglone	n.r	[4]
*D. usambarensis*					
	root	naphthoquinone	7-methyljuglone, isodiospyrin, diosindigo A and B, *bis*-isodiospyrin and mamegakinone	MeOH	[54,80]
	stem bark	naphthoquinone	7-methyljuglone and diosindigo A	MeOH	[54]
*D. verrucosa*					
	root bark	naphthoquinone	diosindigo A, 7-methyljuglone, diosquinone and isodiospyrin	n.r	[57]
	root bark	triterpenoid	betulinic acid and betulin		
	stem bark	naphthoquinone	diosindigo A, 7-methyljuglone, diosquinone and isodiospyrin	n.r	[57]
	stem bark	triterpenoid	betulinic acid and betulin	n.r	[57]
*D. whyteana*					
	n.r	naphthoquinone	7-methyljuglone	n.r	[4]
*D. zombensis*					
	bark	triterpenoid	oleanolic acid	MeOH	[4]
	root bark	naphthoquinone	7-methyljuglone, diosquinone, isodiospyrin and mamegakinona	P. ether, MeOH	[4,61]

Extract: Ace—acetone; CF—chloroform; DCM—dichloromethane; Ee—ether; EtOAc—ethyl acetate; EtOH—ethanol; H_2_O—water; Hex—hexane; MeOH—methanol; P. ether—petroleum ether; n.r—not reported.

**Figure 4:**
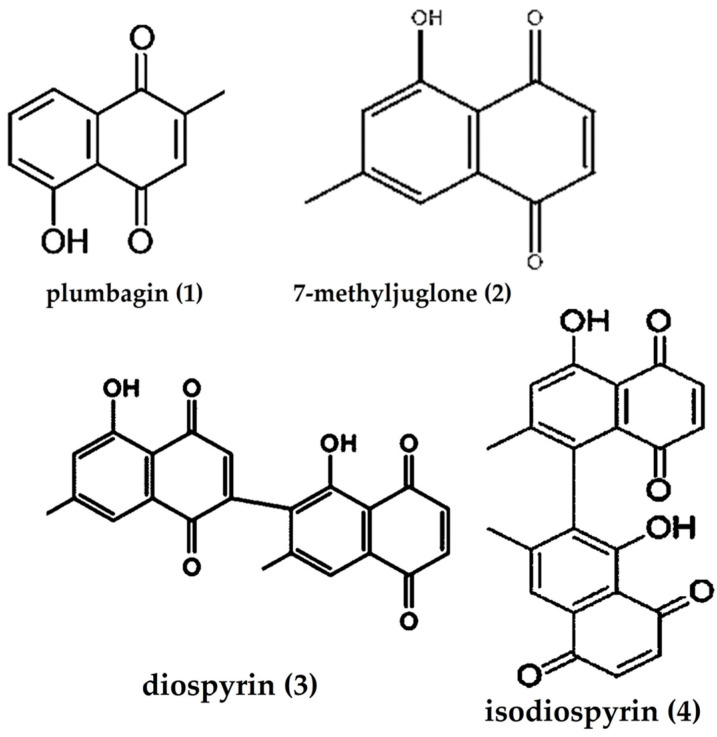
*Diospyros* representatives identified 1,4-naphthoquinones.

Among the NQs (Figure 4), 80% are 1,4-NQs, either as monomers such as plumbagin (**1**) and 7-methyljuglone (**2**) or as dimers such as diospyrin (**3**) and isodiospyrin (**4**), while trimers and tetramers are less represented in this genus [4,81].

In the Mozambican *Diospyros* species, plumbagin (**1**) and 7-methyljuglone (**2**) are the most prominent 1,4-NQs identified [3,4]. The presence of 7-methyljuglone has been reported in diethyl ether, dichloromethane, chloroform, methanol, and hydroethanol extracts of the root, stem, and bark of most species [1,5] and in the ether extract of *D. lycioides* branches [41].

Plumbagin has been identified on the root bark of *D. abyssinica* [28], and isodiospyrin (**4**), a dimeric 7-methyljuglone derivative [3], has been reported in a hexane extract of *D. dichrophylla* seeds [66] and in the diethyl ether extract of bark and phylum of almost all Mozambican *Diospyros* species [4].

*D. mespiliformis* has been one of the best-studied Mozambican *Diospyros species*, having NQs identified in different plant parts [4,79] and triterpenoids in leaf, bark, and stem bark [4,76,82].

**Figure 5:**
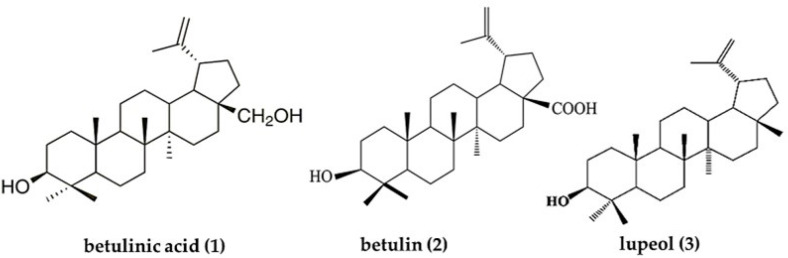
*Diospyros* identified representative lupan-type triterpenoids.

Triterpenoids (lupane, ursane, oleanane, taraxerane, and friedelane) are present in more than 90% of *Diospyros* species. Lupane-type compounds (Figure 5), such as betulinic acid (**1,** Figure 5), betulin (**2**, Figure 5), and lupeol (**3**, Figure 5), are the most active substances present in *Diospyros* African species [4,64,83,84]. These compounds were detected in different types of extracts (petroleum ether, dichloromethane, chloroform, methanol, hydroethanol, and aqueous extracts) and their fractions [1,5,28,41]. Several biological activities have been demonstrated for them, mainly for betulinic acid and its derivatives [83,85,86,87,88].

Condensed tannins (proanthocyanidins and oligopolymeric complex tannins), and particularly hydrolysable tannins (gallotannins, ellagitannins), and have also been identified in Mozambican *Diospyros* species such as *D. villosa* [4,25,58] and *D. mespiliformis* [82,89].

In addition, from the methanolic extract derived from *D. lycioides* twigs, three naphthalene glycosides were identified [72], and carotenoids were identified in the fruit of this species [90]. The presence of galactiol and vitamin E in the *D. ferrea* leaf was also reported [67].

So far, the biologically active marker secondary metabolites isolated and studied from several species of the genus *Diospyros* have mainly been naphthoquinones, triterpenoids, and tannins. Compounds belonging to these chemical classes have been isolated from the twigs, bark, roots, leaves, stems, and fruits of Mozambican species of this genus. Examples include plumbagin, 7-methyljuglone, diospyrin, and isodiospyrin, which have been isolated from the root of several *Diospyros* species.

### 2.3. In Vitro and In Vivo Biological Activity of Mozambican Diospyros Species and Marker Compounds

In Table 4, Table 5 and Table 6, the different in vitro and in vivo biological activities and toxicological tests performed on Mozambican *Diospyros* species, and their isolated marker secondary metabolites are summarized. A total of thirteen species (41.9%), namely *D. abyssinica*, *D. bussei*, *D. ferrea*, *D. kabuyeana*, *D. lycioides*, *D. loureiriana*, *D. mafiensis*, *D. mespiliformis*, *D. natalensis*, *D. squarrosa*, *D. usambarensis*, *D. verrucosa*, and *D. villosa*, were evaluated for biological activities other than antibacterial activities (Table 4).

#### 2.3.1. Anti-Inflammatory and Analgesic Activity

Aqueous extract of *D. abyssinica* root bark has shown stronger anti-inflammatory activity (enzyme 15-lipoxygenase (LOX) inhibition) than quercetin [27].

In vivo assays have shown that the hexane fraction of *D. mespiliformis* leaves has anti-inflammatory properties (inhibits stronger the LOX), and that the methanolic extracts of different plant parts showed wound healing effects. On the other hand, the butanol and ethyl acetate fractions activate LOX activity. These results show that *D. mespiliformis* extract can have pro-inflammatory and anti-inflammatory effects [51].

Lupeol isolated from *D. mespiliformis* stem bark has shown analgesic activity in both pain inhibition (neurological-first phase) and origin (inflammatory-second phase) in biphasic tests (in vivo) [76].

#### 2.3.2. Antihyperglycemic Activity

Another finding has revealed that the oral administration of a methanolic extract obtained from the leaves of *D. ferrea* (400 mg/kg) for a duration of 21 days in diabetic rats showed significant antihyperglycemic activity [91]. The root of this species is rich in phenolic acids, especially gallic acid, and is therefore traditionally used as a potent antioxidant [70].

#### 2.3.3. Antifungal Activity

Several studies have reported the potential antifungal activity of the root and root bark of most *Diospyros* species [42,54,92]. However, the antifungal activity of a leaf extract of *D. mespiliformis* has also been confirmed [47,93].

Various *Diospyros* medicinal plants are also effective against *Candida* spp. [1]. The methanolic extract of the *D. abyssinica* root is active against this microorganism [94]; however, in another study, it was only moderately active against the same microorganism [95]. Another medicinal plant, *D. mespiliformis*, is more active against *C. neoformans* than against *C. albicans*. A leaf extract showed anti-*C. albicans* activity, while a bark extract showed in vitro activity against *C. neoformans*-isolated strains from South African AIDS patients [96].

*D. mespiliformis*, traditionally used to treat ringworm, shows remarkable antimicrobial activity against *Trichophyton mentagrophytes* and *Microsporum canis*. This result supports the traditional use of this species against dermatophytosis [47]. Aqueous and ethanolic extracts of the leaf and bark of *D. mespiliformis* showed significant antifungal activity against *Aspergillus niger*, *Aspergillus flavus*, and *Microsporum gypseum* [97].

#### 2.3.4. Antiparasitic Activity

*Diospyros* species have antiparasitic activity, especially against both chloroquine-sensitive (3D7) and chloroquine-resistant (FcB1) strains of *Plasmodium falciparum* [31,94].

The decoction of the stem of *D. mespiliformis* was tested against *Plasmodium berghei*-infected mice and demonstrated potent activity, including the inhibition of beta-hematin in an in vitro study [98].

In vitro studies from methanolic extracts of *D. abyssinica* leaves have provided confirmation of its antiparasitic activity against *Leishmania donovani* [65,94], *Trypanosoma cruzi*, *Trypanosoma brucei* [99], *Culex*, and *Anopheles* larvae [94].

The isolated compound 7-methyljuglone obtained from the methanolic extract of *D. usambarensis* root bark has significant schistosomicidal activity [54,92].

#### 2.3.5. Antioxidant Activity

The scavenging activity of crude extract and fractions of four *Diospyros* species, namely *D. abyssinica*, *D. lycioides*, *D. mespiliformis*, and *D. villosa*, present in the Mozambican Flora was evaluated spectrophotometrically using the DPPH (1,1-diphenyl-2-picrylhydrazyl) radical assay.

An estimation of the concentration of antioxidant vitamins (i.e., A, C, and E) from crude methanolic extracts obtained from the leaf, bark, and root of *D. mespiliformis* was also determined using the DPPH [51].

**Table 4:** In vitro and in vivo non-antibacterial tests of biological activity in Mozambican species of *Diospyros* and marker compounds.

Biological Activity/ Species	PU	Extract/ Compound	Results	Microorganism/ Assay	Control	Ref.
**Analgesic**						
*D. mespiliformis*	SB	CF/lupeol 25 mg/kg, p.o	Pi^1^ 2.2 ± 0.2/ asa =1.0 ± 0.3 Pi^2^ 1.98 ± 0.1/ asa =1.15 ± 0.1	Biphasic, Wistar rats	acetylsalicylic acid (asa), 100 mg/kg, p.o.	[76]
*D. ferrea*	L	CF MeOH	100–300 mg/Kg significant activity	Tail flick method, adult Wistar albino rats	ibuprofen	[100]
*D. ferrea*	R	CF MeOH	100–200 mg/Kg significant activity	Tail flick method, adult Wistar albino rats	ibuprofen	[101]
**Anti-inflammatory**						
*D. abyssinica*	Rb	H_2_O (1) MeOH (2)	1—IC_50_ = 16 ± 1 μg/mL 2—IC_50_ = 86 ± 7 μg/mL	LOX, using soybean lipoxygenase type 1-B	quercetin, IC_50_ value 11.5 ± 0.6 μg/mL	[27]
*D. ferrea*	L	CF MeOH	100–300 mg/Kg = 26.2–28.2% 100–300 mg/Kg = 29.6–37.6%	PIPE, adult male Wistar rats	ibuprofen 41.1%	[100]
*D. ferrea*	R	CF MeOH	100–200 mg/Kg = 37%	PIPE, adult Wistar albino rats	ibuprofen	[101]
*D. mespiliformis*	Sb	DCM Fraction maximally at 400 mg/kg	Modulation of serum concentrations of Tumour Necrosis Factor alpha and Interleukin 1 beta and 6	Cytokine inhibition, *Plasmodium berghei*-infected mice	artemether-lumefantrine	[98]
	L	Hex Fraction 5 μg/mL(1) 10 μg/mL (2)	1—IC_50_ = 31.21 ± 0.84 μg/mL 2—IC_50_ = 32.05 ± 2.79 μg/mL	LOX, Wistar rats	quercetin, IC_50_ value 1–46.02 ± 5.46 μg/mL 2–32.05 ± 2.79 μg/mL	[51]
**Antihyperglycemic**						
*D. ferrea*	L	MeOH 21 days	400 mg/kg, i.p, significant antihyperglycemic activity	Streptozotocin induced diabetic Wistar rats	glibenclamide, 0.5 mg/Kg, p.o.	[91]
**Antifungal**						
*D. abyssinica*	R	MeOH	Actives in test controlled by conidial suspension	BA, *C. albicans* *C. cucumerinum*	methylthiazolyltetrazolium chloride (MTT)	[94]
*D. ferrea*	W	1-isodiospyrin 2-plumbagin	1—active against three fungi 2—active against eight fungi	HMBC	*Phomopsis* sp. reference spectrum for both H1 and C13	[102]
*D. mafiensis*	Rb	3-hydroxy- diosquinone	MIC_50_ = 14.9 µg/mL MIC_50_ = 39.1 µg/mL	CCA, *A. f*lavus, *A. parasiticus*	*A. parasiticus* B62	[42]
	Rb	3-hydroxy- diosquinone	Reduced total aflatoxin, 1.145 to 32 ng/plac	ELISA, *A. parasiticus*, *A. flavus*	*A. parasiticus* B62	[42]
	Rb	diosquinone	MIC_50_ >100 µg/mL	CCA, *A. flavus*, *A. parasiticus*	*A. parasiticus* B62	[42]
	Rb	diosquinone	Reduced total aflatoxin 1.145 to 45 ng/plac	ELISA, *A. flavus*, *A. parasiticus*	*A. parasiticus* B62	[42]
	Rb	P. ether, DCM (E) Fraction (F)	E = 5 mg/disc IZ: 7–20 mm F = 0.2 mg/disc IZ: 19–20 mm	DD, *C. albicans*	miconazole 20 µg/disc IZ: 29 mm	[103]
*D. mespiliformis*	Rb L	Ace	MIC = 0.16 μg/mL	BD, *C. albicans*, *M. canis*	amphotericin B MIC = 0.02 μg/mL	[93]
	L	DCM:MeOH	MIC = 0.10–0.50 mg/mL	BD, *M. canis*, *T. mentagrophytes*	tetrazolium violet	[47]
	L	H_2_O	MIC = 0.08 μg/mL	BD, *M. canis*	amphotericin B MIC = 0.02 μg/mL	[93]
	B	Ace	IZ: 7 mm (1) IZ: 12 mm (2)	ADD, 1-*C. albicans*, 2-*C. neoformans*	nystatin	[96]
*D. usambarensis*	Rb	7-methyljuglone	MIC = 0.025 μg/mL	BA, *C. cucumerinum*	miconazole MIC = 0.001 μg/mL	[92]
	Rb	isodiospyrin	MIC = 10 μg/mL	BA, *C. cucumerinum*	miconazole MIC = 0.001 μg/mL	[54]
*D. villosa*	R	EtOH 70% Fraction	MIC = 312.5 μg/mL MIC = 62.5–312.5 μg/mL	BD, *C. albicans*	not reported	[104]
**Antiparasitic**						
*D. abyssinica*	L	EtOAc	IC_50_ = 51.3 ± 8.8 μg/mL	BD, *P. falciparum* (FcB1)	chloroquine	[31]
	B	EtOAc	IC_50_ = 1.5 μg/mL	*L. donovani*	pentamidine	[65]
			IC_50_ = 5.6 μg/mL	*P. falciparum*	chloroquine	
	B	diospyrin isodiospyrin	IC_50_ = 0.5 μM	*L. donovani*	pentamidine IC_50_ = 7 μM	[94]
	B	diospyrin isodiospyrin	IC_50_ = 1.5 μM	*P. falciparum* (FcB1)	chloroquine IC_50_ = 0.1 μM	[94]
	R	DCM MeOH	MIC = 500 mg/L	*Culex*, *Anopheles larvae*	not identified	[94]
*D. bussei*	R	MeOH	IC_50_ = 65.7 ± 2.7 μg/mL	*T. brucei* (Lister 427)	pentamidine IC_50_ = 0.000509 μM	[99]
*D. kabuyeana*	L	MeOH	IC_50_ = 3.32 μg/mL	*T. brucei* (Lister 427)	pentamidine IC_50_ = 0.000509 μM	[99]
*D. loureiriana*	Rb Sb L	MeOH	IC_50_ = 1.68 ± 0.77 μg/mL IC_50_ = 11.53 ± 1.99 μg/mL IC_50_ = 19.10 ± 4.41 μg/mL	*P. falciparum* (3D7)	chloroquine IC_50_ = 0. 0045 μM	[105]
						[105]
						[105]
*D. mespiliformis*	S	EtoAC (1) DCM (2) MeOHfraction (3)	1—IC_50_ = 3.18 μg/mL 2—IC_50_ = 0.78 μg/mL 3—IC_50_ = 0.55 μg/mL	*Plasmodium berghei*-infected mice	artesunate and chloroquine diphosphate	[98]
*D. natalensis*	Sb	MeOH	IC_50_ = 2.85 μg/mL	*T. brucei* (Lister 427)	pentamidine IC_50_ = 0.000509 μM	[99]
*D. squarrosa*	Rb	MeOH	IC_50_ = 5.38 μg/mL	*T. brucei* (Lister 427)	pentamidine IC_50_ = 0.000509 μM	[99]
*D. verrucosa*	Sb	MeOH	IC_50_ = 1.28 μg/mL	*T. brucei* (Lister 427)	pentamidine IC_50_ = 0.000509 μM	[99]
	R	MeOH	IC_50_ = 2.23 μg/mL	*T. brucei* (Lister 427)		
	L	MeOH	IC_50_ = 2.99 μg/mL	*T. brucei* (Lister 427)		
*D. usambarensis*	Rb	7-methyljuglone	Efficiency schistosomiasis MIC = 5 ppm	*Biomphalaria glabrata*	not identified	[54]
**Antioxidant**						
*D. abyssinica*	Rb	EtOH (1) MeOH (2) H_2_O (3)	1-EC_50_ = 16.0 ± 2 μg/mL 2-EC_50_ = 16.6 ± 0.4 μg/mL 3-EC_50_ = 21 and 29 ± 2 μg/mL	DPPH	quercetin EC_50_ value 3.4 ± 0.3 μg/mL	[27]
*D. lycioides*	L	Ace	𝑅𝑓 = 0.54; 0.60; 0.83; 0.89	DPPH on TLC plates	phenolic compounds	[38]
*D. mespiliformis*	F	MeOH	87.36% at 1 mg/mL	DPPH	vitamin E	[106]
	R	MeOH	IC_50_ = 3.47 ± 0.05 μg/mL	DPPH	ascorbic acid 2.36 ± 0.30 μg/mL trolox 3.43 ± 0.78 μg/mL	[51]
	F	MeOH	IC_50_ = 6.94 ± 0.49 μg/mL	DPPH		[51]
	B	MeOH	IC_50_ = 7.82 ± 0.76 μg/mL	DPPH		[51]
	L	EtOAc Fraction	IC_50_ = 1.08 ± 0.04 μg/ml	DPPH	ascorbic acid 5.08 ± 0.12 μg/mL	[51]
*D. villosa*	Sb	MeOH	IC_50_ = 9.53 μg/mL	DPPH	ascorbic acid 10.3 μg/mL	[107]
	L	CF (1) Hex (2)	1-IC_50_ = 10.7 μg/mL 2-IC_50_ = 11.8 μg/mL	DPPH	ascorbic acid 10.3 μg/mL	[107]

Part used (PU): L—leaf; B—bark; F—fruit; R—root; Rb—root bark; Sb—stem bark. Extract: Ace—acetone; ADD—agar disc diffusion; CF—chloroform; DCM—dichloromethane; EtOAc—ethyl acetate; EtOH—ethanol; H_2_O—water; Hex—hexane; MeOH—methanol; P. ether—petroleum ether. Test: BA—TLC bioautography; BD—broth dilution; CCA—cell culture in agar; DD: disco diffusion method; DPPH—2,2-diphenyl-1-picrylhydrazyl; ELISA—enzyme-linked immunosorbent assay; HMBC—heteronuclear multiple-bond correlation method; PIPE—percent inhibition of paw edema. Abbreviations: LOX-15-lipoxygenase; Pi**^1^**—pain inhibition (neurological-first phase); Pi^2^—pain inhibition (inflammatory-second phase); EC50—half maximal effective concentration; IC50—half maximal inhibitory concentration; MIC—minimum inhibitory concentration.

#### 2.3.6. Cytotoxicity, Genotoxicity, and Toxicity of Mozambican *Diospyros* Species

The results of in vitro cytotoxicity tests using normal and tumorous human cells and *Artemia salina*, as well as in vitro genotoxicity and in vivo acute and sub-chronic toxicity assessment of *Diospyros* species, are summarized in Table 5.

**Table 5:** In vitro cytotoxicity and genotoxicity studies as well as in vivo toxicity studies in Mozambican *Diospyros* species.

Species	Parts Used	Extract	Toxicity Assay	Results	Ref.
*D. abyssinica*					
	leaf	EtOAc	Cytotoxicity against MRC-5 human diploid embryonic cells, Taxotere^®^ as standard	IC_50_ = 6.0 ± 5.0 μg/mL	[31]
	leaf	EtOAc	Cytotoxicity against KB human tumor cell lines (squamous cell carcinoma of the mouth), Taxotere^®^ as standard	>85% cell inhibition IC_50_ = 1.0 ± 2.0 μg/mL	[31]
	bark	EtOAc	Cytotoxicity against human KB cell (1) and *Rhabditis pseudoelongata* (2)	(1) LD_50_ = 10 μg/mL (2) LD_50_ = 1 μg/mL	[65]
*D. dichhropylla*					
	seed	DCM:MeOH (1) isodiospyrin (2)	Cytotoxicity using Brine shrimp test (*Artemia salina*)	1-(LC_50_ = 29 μg/mL) 2-(LC_50_ = 0.13 μg/mL)	[66]
*D. ferrea*					
	leaf	MeOH	In vivo—acute oral toxicity using male Wistar albino rats	LD_50_ = 2000 mg/kg	[91]
	fruit	isodiospyrin (1) 8′-hydroxyisodiospyrin (2)	Cytotoxicity strong against Hep-3B, KB, COLO-205, and HeLa cancer cells	1(ED_50_ = 0.17, 1.72, 0.16 and 0.21 μg/mL) 2(ED_50_ = 1.31, 1.75, 1.96 and 1.79 μg/mL)	[68]
*D. lycioides*					
	leaf	Ace	Cytotoxicity against BUD-8 cell (human fibroblast cells) in real-time xCELLigence system and 7.4 μg/mL curcumin (control)	IC_50_ = 500 and 1000 μg/mL	[38]
	leaf	Ace	Cytotoxicity against HeLa cells mobility assayed using the wound healing assay and 7.4 μg/mL curcumin (control)	Nontoxic to the normal cell at 300 μg/mL	[38]
*D. loureiriana*					
	root bark	MeOH	Cytotoxicity against human embryonic kidney cells (HEK293), estimated growth inhibition at 400 μg/ml	IC_50_ = 100.34 ± 9.85 μg/mL	[105]
	stem bark			IC_50_ = 57.26 ± 0.53 μg/mL	[105]
*D. mafiensis*					
	root bark	P. ether (1) DCM (2) EtOH (3) fraction P. ether (4) fraction DCM (5)	Cytotoxicity using brine shrimp larvae test (*Artemia salina*) Standard cyclophosphamide LC_50_ value of 17.78 µg/mL	1-LC_50_ = 25.12 µg/mL 2-LC_50_ = 69.18 µg/mL 3-LC_50_ = 120.23 µg/mL 4-LC_50_ ≤ 8–45.71 µg/mL 5-LC_50_ = 5.08 µg/mL	[103]
*D. mespiliformis*					
	stem bark root bark	EtOH	In vivo—acute oral toxicity using Wistar rats of both sexes	LD_50_ = 570 mg/kg Acute toxicity is moderate	[49]
	leaf	MeOH	in vivo—acute oral administration using rats	LD_50_ ≥ 5 g/kg	[108,109]
	stem bark				
	leaf	EtOAc fraction	In vivo—sub-chronic toxicity using rats	LD_50_ = 750 g/kg	[108]
	stem bark			LD_50_ = 500 g/kg	
	root	diosquinone	Cytotoxicity against human glioblastoma cell lines (1) and hormone-dependent human prostate cancer (2)	1-ED_50_ = 0.18 μg/mL 2-ED_50_ = 4.50 μg/mL	[84]
*D. whyteana*					
	twigs	DCM	Genotoxicity against mutagens mitomycin C (MMC) using the Ames test (*Salmonella typhimurium* TA98)	protective effect non-genotoxic at 500–2500 μg/mL	[60]
	leaf	DCM HydroMeOH 90%	Genotoxicity using the Ames test (*Salmonella typhimurium* TA98)	shift mutations of lowest dose is 0.50 μg/mL higher doses are toxic	[110]
*D. villosa*					
	root	HydroEtOH 70%	In vivo—acute toxicity using mice	possible renal dysfunction development	[58]
*D. zombensis*					
	root bark	7-methyljuglone (1) isodiospyrin (2)	Cytotoxicity against human colon carcinoma cells	1-LD_50_ of 7.0 × 10^−2^ μg/mL 2-LD_50_ of 3.8 × 10^−2^ μg/mL	[61]

Extracts: Ace—acetone; DCM—dichloromethane; EtOAc—ethyl acetate; EtOH—ethanol; H_2_O—water; Hex—hexane; HydroEtOH—ethanol; HydroMeOH—methanol; MeOH—methanol; P. ether—petroleum ether. Concentration: ED_50_—median effective dose; IC_50_—half maximal inhibitory concentration; LC_50_—lethal concentration 50%, LD_50_—lethal dose 50%.

Most commonly, studies were found to be related to the in vitro assessment of cytotoxicity. For example, the extract of *D. lycioides* showed cytotoxicity to HeLa cells but was non-toxic to normal cells [38]. The compound diosquinone has been shown to be toxic against most cancer cell lines (human glioblastoma) and hormone-dependent human prostate cancer [84]. In contrast, 7-methyljuglone and isodiospyrin compounds are active against human colon carcinoma cells [61].

**Figure 6:**
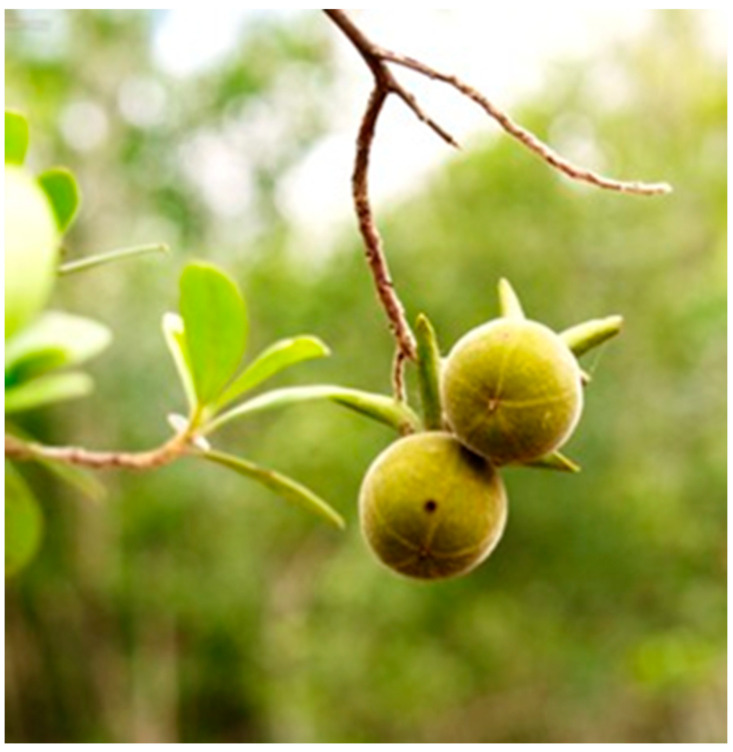
*Diospyros dichrophylla* (Gand.) De Winter: Detail of fruits in nature, Mandevo, Namaacha district, Maputo, 2010. Photography by Elsa Gomes.

The organic extract of the inner seed of *D. dichrophylla* (Figure 6) is reported as highly cytotoxic (LC_50_ = 29 μg/mL), particularly the isodiospyrin isolated from it (LC_50_ = 0.13 μg/mL) [66].

Preclinical safety assessments of *Diospyros* species are of paramount importance; however, few studies related to Mozambican *Diospyros* species have been conducted to date. Cantrell et al. (2003) reported that *D. dichrophylla* is a potent phytotoxicant due to the presence of isodiospyrin (from the inner seed) at a lethal dose of 0.13 g/mL [66]. In another study, a hydroethanolic root extract of *D. villosa* showed possible development of renal dysfunction using an acute toxicity test in mice [111].

#### 2.3.7. Antibacterial Activity

In vitro antibacterial activity data collected from eleven *Diospyros* species (representing 35.5% of the total) are summarized in Table 6. Of the 11 species examined, 47 extracts (including AgNPs) showed antimicrobial activity against multiple bacterial strains. The methanolic extract was the most tested. In some of the studies mentioned, biodirected fractionation was also performed, and the antibacterial activity of the obtained fractions and isolated compounds was determined. The results obtained are also shown in Table 6.

**Table 6:** In vitro antibacterial activity of Mozambican *Diospyros* and marker compounds.

Species	Parts Used	Test	Extract/ Compound	MIC (μg/mL)	Microorganism	Control (MIC) μg/mL	Ref.
*D. abyssinica*							
	bark	BD	EtOAc	12	*S. aureus* ATCC 6538	DMSO	[65]
*D. bussei*							
	leaf stem bark	BD	MeOH	125	*E. coli* ATCC 8740	ciprofloxacin 0.63	[99]
	leaf	BD	MeOH	8000	*S. aureus* ATCC 25923	ciprofloxacin 2.5	
					*B. cereus* ATCC 11775	ciprofloxacin 0.08	
	root bark	BD	MeOH	500	*E. coli* ATCC 8740	ciprofloxacin 0.63	
*D. kabuyeana*							
	leaf	BD	MeOH	8000	*S. aureus* ATCC 25923	ciprofloxacin 2.5	[99]
	leaf	BD	MeOH	4000	*B. cereus* ATCC 11775	ciprofloxacin 0.08	
	stem bark	BD	MeOH	1000			
	leaf stem bark	BD	MeOH	125	*E. coli* ATCC 8740	ciprofloxacin 0.63	
*D. lycioides*							
	branche	BD	MeOH	1250	*S. sanguis*, *P. gingivalis*, *S. mutans*, *P. intermedia*	alkaloid sanguinarine	[41]
	branche	BD	Diospyroside A	39	*S. sanguis*, *P. intermedia*	alkaloid sanguinarine	[41]
				78–1250	*P. gingivalis*, *S. mutans*		
	branche	BD	Diospyroside B	39–78	*S. sanguis*, *P. gingivalis*	alkaloid sanguinarine	[41]
				156–625	*P. intermedia*, *S. mutans*		
	branche	BD	Diospyroside C	39–156	*P. intermedia*, *S. mutans*	alkaloid sanguinarine	[41]
				312–625	*P. gingivalis*, *S*, *sanguis*		
	branche	BD	Diospyroside D	156–312	*S. mutans*, *P. intermedia*, *P. gingivalis*, *S. sanguis*	alkaloid sanguinarine	[41]
	branche	BD	juglone	19–78	*P. intermedia*, *S. mutans*,	alkaloid sanguinarine	[41]
				39	*S. sanguis*, *P. gingivalis*		
	branche	BD	7-methyljuglone	39–156	*P. gingivalis*, *S. mutans*	alkaloid sanguinarine	[41]
				78	*S. sanguis*, *P. intermedia*		
	leaf	BA	EtOAc	0.10–0.16 *	*P. aeruginosa * ATCC 27853	p-iodonitrotetrazolium chloride	[38]
			Ace	0.12–0.17 *			
	leaf	BA	EtOAc	0.16–0.36 *	*S. aureus * ATCC 29213	p-iodonitrotetrazolium chloride	[38]
			Ace	0.20–0.45 *			
			MeOH	0.16–0.27 *			
	leaf	BA	EtOAc	0.05–0.45 *	*E. faecalis * ATCC 29212	p-iodonitrotetrazolium chloride	[38]
			Ace	0.05–0.45 *			
			MeOH	0.05–0.45 *			
*D. mafiensis*							
	root bark		DCM	*S. aureus* *B. anthracis* IZ: 12 mm	*S. typhi*, *S. boydii*, *E. coli*, *K. pneumoniae* *S. aureus*, *V. cholerae* *Proteus sp.*, *B. anthracis*	gentamycin ampicillin (20 µg/disc)	[103]
	root bark		P. ether-Fraction	IZ: 10–15 mm	*S. typhi*, *S. boydii*, *E. coli*, *K. pneumoniae* *S. aureus*, *V. cholerae* *Proteus* sp., *B. anthracis*	gentamycin ampicillin (20 µg/disc)	[103]
*D. mespiliformis*							
	leaf	ADD	MeOH	167	*S. aureus*	isoniazid 5.0	[50]
	root	ADD	MeOH	250	*S. aureus*	isoniazid 5.0	[50]
	leaf	BD	EtOH	12,500–25,000	*Salmonella* spp., *Shigella* spp., *Campylobacter* spp.	ciprofloxacin, cefixime, and gentamicin	[95]
	leaf	BD	Hex (F1) nBOH (F2) EtOAc (F3) H_2_O (F4)	(1)78.125–312.5 (2)156.25 (3)78.125–156.25 (4)625–2500	1*-P. aeroginosa* 2*-S. aureus* 3*-E. coli* 4-*S. typhimurium*	gentamicin 19.53 gentamicin 19.53 gentamicin 19.53 gentamicin 19.53	[97]
	leaf			625			
	root			625 (1) >2500 (2 to 4)			
	leaf	AD	H_2_O HydroMeOH 10%	250–500 125–500	*H. influenzae* (6 ci)	ampicillin 0.12–15.6	[112]
			H_2_O HydroMeOH 10%	125–250 62.5–125	*S. aureus* (5 ci)	ampicillin 0.06–0.12	
			H_2_O HydroMeOH 10%	250–250 125–125	*S. pneumoniae* (3 ci)	ampicillin 0.015–0.12	
			H_2_O HydroMeOH 10%	250–250 125–125	*S. pyogenes* (8 ci)	ampicillin 0.015–0.06	
			H_2_O HydroMeOH 10%	250–500 125–250	*M. catarrhalis* (5 ci)	ampicillin 0.12–1.9	
	leaf	BD	flavonol O-rhamnoside	9770	*S. aureus*	not identified	[77]
	root	AD	diosquinone	3–30	*S. aureus* NCT 6571 *S. aureus* E3T	ampicillin 5	[78]
			diosquinone	15–16	*E. coli* KL16 *P. aeruginosa* NCT 6750	gentamicin 2	
	leaf	DD	methylated flavone	IZ: 34 mm	*E. coli*	not identified	[78]
	leaf	AWD	EtOH-Fraction	IZ: 20 mm IZ: 18 mm IZ: 16 mm	*S. aureus*, *Shigella* spp. *P. aeruginosa*	septrin 15 mm spetrin 16 mm spetrin 15 mm	[113]
*D. natalensis*							
	leaf	BD	MeOH	250	*S. aureus* ATCC 25923	ciprofloxacin 0.08	[99]
	leaf		MeOH	1000	*B. cereus* ATCC 11775	ciprofloxacin 2.5	
	leaf		MeOH	500	*E. coli* ATCC 8740	ciprofloxacin 0.63	
	root bark		MeOH	1000	*E. coli* ATCC 8740	ciprofloxacin 0.63	
	stem bark		MeOH	250	*E. coli* ATCC 8740	ciprofloxacin 0.63	
*D. rotundifolia*							
	not reported		Ace	230–1770	*S. aureus*, *E. faecalis*, *E. coli* and *P. aeruginosa*	not reported	[22]
*D. squarrosa*							
	leaf	BD	MeOH	4000	*B. cereus* ATCC 11775	ciprofloxacin 2.5	[99]
			MeOH	250	*E. coli* ATCC 8740	ciprofloxacin 0.63	
	root bark	BD	MeOH	1000	*S. aureus* ATCC 25923	ciprofloxacin 0.08	
			MeOH	4000	*B. cereus* ATCC 11775	ciprofloxacin 2.5	
	stem bark	BD	MeOH	500	*E. coli* ATCC 8740	ciprofloxacin 0.63	
*D. verrucosa*							
	leaf	BD	MeOH	1000	*S. aureus* ATCC 25923	ciprofloxacin 0.08	[99]
			MeOH	2000	*B. cereus* ATCC 11775	ciprofloxacin 2.5	
			MeOH	500	*E. coli* ATCC 8740	ciprofloxacin 0.63	
	root bark stem bark	BD	MeOH	<6.25	*E. coli* ATCC 8740	ciprofloxacin 0.63	[82]
*D. villosa*							
	root	BD	HydroEtOH 70% Ee Fractions	62.5–312.5 15.6–62.5 31.2–62.5	*E. faecalis* ATCC 435628	not reported	[104]
					*E. coli* ATCC 25922	not reported	
					*M. luteus* ATCC 10240	not reported	
					*S. aureus* ATCC 25923	not reported	
	leaf	DD	AgNPs	IZ: 15 mm	*E. coli* ATCC 25922	ciprofloxacin 37 mm gentamicin 20 mm	[107]
			AgNPs 80 °C	IZ: 18 mm	*S. aureus* ATCC 700698	ciprofloxacin 6 mm gentamicin 11 mm	
			AgNPs	IZ: 16 mm	*S. epidermidis * ATCC 12228		
			AgNPs	IZ: 16 mm		ciprofloxacin 28 mm gentamicin 20	
			Ace	0.05–0.45 *			
			MeOH	0.05–0.45 *			

Test: BD—broth dilution; DD—disc diffusion; ADD—agar disc diffusion; AWD—agar well diffusion. Extract: Ace—acetone; DCM—dichloromethane; Ee—ether; EtOAc—ethyl acetate; EtOH—ethanol; H_2_O—water; Hex—hexane; HydroMeOH—methanol; nBOH—n-butanol; P. ether—petroleum ether. Strains: *B. anthracis*—*Bacillus anthracis*; *B. cereus*—*Bacillus cereus*; *E. faecalis*—*Enterococcus faecalis*; *H. influenzae*—*Haemophilus influenzae*; *K. pneumoniae*—*Klebsiella pneumoniae*; *M. catarrhalis*—*Moraxella catarrhalis*; *M. luteus*—*Micrococcus luteus*; *P. gingivalis*—*Porphyromonas gingivalis*; *P. intermedia*—*Prevotella intermedia*; *S. typhi*—*Salmonella typhi*; *S. typhimurium*—*Salmonella typhimurium*; *S. boydii*—*Shigella boydii*; *S. epidermis*—*Staphylococcus epidermidis*; *S. sanguis*—*Streptococcus sanguinis*; *S. mutans*—*Streptococcus mutans*; *S. pyogenes*—*Streptococcus pyogenes*; *V. cholerae*—*Vibrio cholerae*. Abbreviations: ATCC—American type culture collection, BA—TLC bioautography, ci—clinical isolate; IZ—zone of inhibition; MIC—minimum inhibitory concentration; AgNPs—silver nanoparticles; * Rf—retardation factor.

According to the WHO, oral diseases are the most common non-communicable diseases, affecting people throughout life and causing pain, discomfort, disfigurement, and even death [114]. The Global Burden of Disease Study reports that oral diseases are among the leading causes of health problems, estimating that half of the world’s population is affected by these diseases [114,115]. The same study provided a comprehensive assessment, and among the results evaluated, permanent tooth decay was the most common cause, representing a major public health problem in many countries [116]. Therefore, preventing and controlling the spread of this health problem is a global challenge, requiring greater efforts and potentially innovative approaches to achieve it. The branches of several *Diospyros* (particularly *D. lycioides*, *D mespiliformis*, and *D. villosa)* are used as toothbrushes for oral care [41,44,52,104,117], and their plant extracts have been shown to be effective against common oral pathogens, including *Streptococcus mutans*, *S. sanguis*, periodontal pathogens (*Porphyromonas gingivalis* and *Prevotella intermedia*), *Lactobacillus* spp., and several strains of *Candida* spp. [41,44,52,104,117]. In fact, over the past few decades, the scientific community has become increasingly interested in understanding the versatility of medicinal plants from traditional herbal medicine and their guaranteed availability to improve clinical approaches to infectious diseases with the intention of reducing antimicrobial resistance [4].

### 2.4. Secondary Metabolites of Mozambican Diospyros Species as Potential Antimicrobial Agents

#### 2.4.1. Naphtoquinones

##### Antibacterial Activity

Plumbagin (**1**, Figure 4) is recognized as an effective antibacterial agent against both Gram-positive and Gram-negative strains of bacteria. This compound has also shown significant inhibitory activity (MIC < 12.5 μg/mL) against the resistant strain of Mycobacterium tuberculosis H37Rv [3,78,118]. Plumbagin isolated from the bark extract of D. maritima and showed activity against S. aureus and Aeromonas hydrophila (MIC = 0.625 and 5 µg/mL, respectively) [119]. In addition, it has also been obtained from the root of D. mespiliformis and has been described as one of the active marker compounds as well as an effective antibacterial agent against Gram-positive and Gram-negative bacterial strains [50,77,112].

Another important compound isolated from *D. hebecarpa*, 7-methyljuglone (**2**, Figure 4), also present in the root of *Euclea natalensis* (*Ebenaceae*), is potentially active against *Mycobacterium tuberculosis* (H37Rv) [18].

Isodiospyrin (**4**, Figure 4), a dimeric 7-methyljuglone-derivative, has been reported to be more active than diospyrin (**3**, Figure 4) against various Gram-positive strains, including *Streptococcus pyogenes*, *S. pneumoniae*, *Corynebacterium diphtheriae*, *Bacillus subtilis*, *Listeria monocytogenes*, *Mycobacterium chelonae*, and *Micrococcus luteus*. Isodiospyrin demonstrates MIC values ranging from (0.78 to 50 μg/mL), while diospyrin shows MIC values ranging from (1.56 to 100 μg/mL) [17].

Extensive research has unveiled the mechanism of action of diospyrin and 7-methyljuglone against *M. tuberculosis*, highlighting their crucial role as non-competitive ATPase inhibitors in key enzymatic reactions [120]. Additionally, emerging evidence has demonstrated the anti-tuberculosis potential of other compounds, such as crassiflorone and plumbagin from *D. crassiflora*, as well as diospyrone and plumbagin from *D. canaliculata*, both derived from the stem bark [121].

In a study conducted by Kuete et al. (2010), it was demonstrated that isobavacalcone and diospirone, derived from *D. canaliculata*, show promise as potential drugs against multidrug-resistant Gram-negative strains. These compounds exhibited enhanced activity when used in combination with efflux pump inhibitors, resulting in MIC values decreased to <10 μg/mL [122,123].

##### Antifungal and Antiviral Activities

The NQs have been well established, particularly against several species of *Candida*, infectious fungi of the mucosa, deep tissues, and the most common fungal diseases in HIV/AIDS patients [124]. Plumbagin inhibits the growth of *C. albicans*, *C. tropicalis*, and other fungi. In addition, fractions derived from plumbagin of *Diospyros* extracts are active against *C. albicans* [1]. In comparison with ketoconazole, a standard antifungal compound, plumbagin is considered a promising antifungal agent and has been used against *C. albicans*, *C. glabrata*, *C. krusei*, *C. tropicalis*, *Cryptococcus neoformans*, *Aspergillus niger*, *A. flavus*, *Alternaria* sp., *Cladosporium* sp., *Geotrichum candidum*, *Fusarium* sp., *Helminthosporum* sp., *M. gypseum*, and *Penicillium* sp. [125,126,127]. This compound, isolated from the stem bark of *D. bipindensis*, also exhibits significant activity against *C. albicans* [128,129,130].

Isolated from the root of *D. virginiana*, 7-methyljuglone and isodiospyrin have significant antifungal activity against *Phomopsis obscurans* (leaf blight), with 97.0% and 81.4% growth inhibition at 30 μM, respectively. These compounds also demonstrate activity against the pathogen *Phomopsis viticola*, with growth inhibition rates of 53.4% and 57.7%, respectively [131].

The antiaflatoxigenic activity of *D. mafiensis* root, another Mozambican medicinal plant, has been linked to the presence of diosquione and 3-hydroxydioquinone, making this herbal drug also an important natural antifungal for preventing fungal growth and aflatoxin accumulation in food [42]. In addition, this species has also been found to have analgesic, antidiabetic, anti-inflammatory, and antioxidant effects, likely correlated with the presence of these kind of constituents.

##### Antiparasitic Activity

NQs are highly active against pathogens in neglected tropical diseases, including malaria, leishmaniasis, and trypanosomiasis (sleeping sickness). Studies examining *Plasmodium* sp. have shown that isodiospyrin-derived isodiospyrol A exhibits antimalarial activity (IC_50_ = 2.7 μg/mL) [132]. Anti-plasmodial activity has also been reported in the ethanolic extract of leafs of *D. monbuttensis* (IC_50_ = 3.2 nM) [133]. Studies on malaria have proposed a redox cycling mechanism (described for the novel antimalarial–antiparasitic drug atovaquone) to support the in vitro activity of diospyrin and its analogues isolated from *D. montana* against *L. donovani* [134].

Plumbagin and its derivative was shown to be active against *Leishmania* spp., while diospyrin was active against *Leishmania donovani* [87]. Semisynthetic crassiflorone derivatives display trypanocidal activity against *T. brucei* and *T. cruzi* [135]. Antiplasmodial activities with IC_50_ values of 16.5 to 29.4 g/mL against chloroquine-sensitive (3D7) and chloroquine-resistant (K1) strains of *P. falciparum* were observed for the juglone-based 1,4-NQs present in *D. sylvatica* [136].

Concerning the assessment of anthelmintic activity, it was demonstrated in vitro that *D. oocarpa*, *D. nigrisence*, *D. candolleana*, and *D. montana* are active on adult earthworms of *Pheritima posthuma* [137]. Similarly, NQ derivatives, including diospyrin from *D. oocarpa*, *D. nigrisence*, and *D. candolleana*, are antiprotozoal in addition to possessing anthelmintic constituents [138].

#### 2.4.2. Triterpenoids

##### Antibacterial and Antifungal Activities

Betulinic acid isolated from the root of *D. lotus* presents a broad spectrum against several Gram-positive and Gram-negative bacteria [85,139,140,141]. Betulin isolated from *D. rubra* is an active agent against *Streptococcus pyogenes*, with a MIC of 85 µg/mL, and *Corynebacterium diphtheriae*, with a MIC range of 64 to 256 µg/mL [88].

Methanolic extract obtained from *D. peregrina* bark and seed containing triterpenoids has been studied for its antidiarrheal properties [142]. Similarly, the methanolic extract of *D. peregrina* fruit showed high activity against *E. coli* (12.6 mm zone of inhibition) and against fungi *C. albicans* (10.7 mm zone of inhibition) and *Penicillium* spp. (7.33 mm) [143].

Betulin present in the hexane fraction isolated from the bark of *D. paniculata* is very efficient against *S. dysenteriae*, which is responsible for diarrhea (MIC = 30 μg/mL) [144]. However, a study of a reductive green synthesis of nano-sized Ag particles using methanolic root extracts of *D. paniculata* showed that the maximum activity was displayed against Gram-positive bacteria compared to Gram-negative bacteria. The maximum activity was observed against *Penicillium notatum*, *A. flavus*, and *Saccharomyces cerevisiae*, with moderate activity towards *C. albicans* and *A. niger* [145].

In another study of ursane-type triterpenoids obtained from the leaf of *D. dendo* Welw. Ex Hiern [EtOH−EtOAc (50:50) extract], antimicrobial activity (62% at 10 μg/mL) against *Pseudomonas aeruginosa* was observed. This Gram-negative bacterium is considered one of the three main causes of human opportunistic infections and has recently been a useful model for the study of biofilm formation, implying antimicrobial resistance to antibiotics [146].

##### Antiviral Activity

Structure–activity relationships between betulinic acid and its synthetic derivatives inhibiting HIV-1 replication, HIV-1 entry, and HIV-protease or reverse transcriptase (RT) have been verified [147,148]. Betulinic acid was identified as a highly promising antiviral (anti-dengue) present in high proportions in most extracts of distinct species of *Diospyros*, particularly from the bark of *D. glans* [83]. Aridanin, isolated from methanol extracts obtained from the leaf, stem, and root of *D. conocarpa*, presents anti-HIV-1IN activity [149].

In a recent study, the antiviral activity of *D. anisandra* was demonstrated against the influenza virus AH1N1pdm09. The *n*-hexane fruit extract exhibited HA inhibitory (HAI) activity, and a fraction of it inhibited the hemagglutination from 12.5 up to 100 μg/mL, which was attributed to the synergistic effect of the different compounds present [150]. Previously, possible antiviral activity against influenza A and B viruses has been attributed to a redox effect of isolated zeylanone epoxide [151].

##### Antiparasitic Activity

Using in vitro antimalarial assays, betulinic acid 3-caffeate isolated from the dried leaf, twig, and branch of *D. quaesita* was shown to be moderately active against both chloroquine-sensitive and chloroquine-resistant *P. falciparum* clones [86]. Lupeol and lupenone, isolated from the dichloromethane and ethyl acetate extracts of *D. rubra* stem, have shown moderate antimalarial activity against *P. falciparum* [88]. On the other hand, hydroethanolic extracts from the trunk of *D. gracilescens* and the hexane fraction showed higher activity against promastigote and amastigote forms of *L. donovani* (IC_50_ = 5.84 μg/mL and IC_50_ = 0.79 μg/mL, respectively) [87]. Aridanin isolated from methanol extracts of the leaf, stem, and root of *D. conocarpa* can be sources of new antitrypanosomal active principles [149].

#### 2.4.3. Tannins

Tannins isolated from Mozambican *Diospyros* species represent an important class of secondary metabolites with remarkable antimicrobial potential against fungi, bacteria, and yeast [152]. Their mechanism of action involves the disruption of microbial enzymes and cell membranes, although their activities are diverse [153]. In addition, recent research has suggested the ability of tannins to generate hydrogen peroxide, which contributes to their important antibacterial properties [154].

##### Antibacterial and Antifungal Activities

*D. melanoxylon* bark is another medicinal plant considered to be active against Gram-positive and Gram-negative bacteria, which is traditionally used for diarrhea, urinary, and skin troubles and has confirmed claims against *E. coli*, *S. aureus*, *S. epidermidis*, *Shigella flexneri*, *Bacillus licheniformis*, *Bacillus brevis*, *Vibrio cholerae*, *P. aeruginosa*, *Streptococcus aureus*, *Candida kruesi*, and *Bacillus subtilis* [155]. Furthermore, it shows promise in the treatment of candidiasis caused by different *Candida* species (*C. viz*, *C. albicans*, *C. krusei*, *C. parapsilosis*, and *C. tropicalis*), with MIC values ranging from 0.375 to 6.0 mg/mL [156]. Extracts derived from the bark of *D. melanoxylon* are rich in tannins and possess significant potential as antimicrobial agents. In a recent study using strains isolated from humans, it was effective against both Gram-positive and Gram-negative bacteria, suggesting the presence of a broad spectrum of antibiotic compounds or simply general metabolic toxins in the plant methanolic extract [157,158]. In another study conducted in India, acetone ethyl acetate and methanol extracts of *D. melanoxylon* showed a MIC < 30 μg/mL against *Aeromonas hydrophila*, *Enterobacter aerogenes*, *E. coli*, and *Klebsiella pneumoniae* [159].

Methanol extract obtained from the bark or seed of *D. peregrina*, which is rich in tannins and other phenols, was evaluated for its antibacterial potential against the pathogenic bacteria associated with diarrhea. The bark extract demonstrated inhibitory effects against *S. aureus*, *Shigella dysenteriae*, *E. coli*, and *P. aeruginosa*, while the seed extract inhibited all tested strains except for *P. aeruginosa* [160]. Similarly, the methanol extract of *D. tricolor* leaves, known for its abundance of tannins and other phenols, exhibited antibacterial activity against both Gram-positive bacteria (*Bacillus cereus* and *S. aureus*) and Gram-negative bacteria (*Salmonella typhii* and *Escherichia coli*) [161].

*Diospyros kaki* Thunb., known as the persimmon tree, is originally from Asia, but it is cultivated in various parts of the world, including Mozambique. Different plant parts are well-known and useful as medicinal plants, and the fruit is known as persimmon. This species has been extensively studied, particularly regarding the antimicrobial activity of the tannins isolated from it. In a study conducted by Liu et al. (2019), the antimicrobial effects of persimmon tannins (PTs) extracted from the fruit of *D. kaki* against methicillin-resistant *Staphylococcus aureus* (MRSA) were investigated. The persimmon tannins (MIC = 1000 μg/mL) displayed potential mechanisms of inhibitory activity (i.e., the tannins can change the normal morphology of MRSA and cause severe damage to the cell wall and cell membrane) [152]. In addition, the hydrolysate of condensed tannins (composed of a polymer of flavan-3-ols, such as catechin groups) exhibited high bacteriostatic activity in vitro against the *M. avium* complex (nontuberculous mycobacteria) that causes opportunistic chronic pulmonary infections [63]. Aqueous extract from the *D. kaki* fruit was tested in vivo, showing interesting antibacterial activities against Gram-negative strains compared to Gram-positive bacteria, justifying its use in traditional medicine for the treatment and/or management of disorders of the digestive system such as diarrhea [162]. The results of another study showed that the condensed tannins extracted from the unripened fruit of *D. kaki* displayed antibacterial activity against biofilms containing multiple bacteria. It is estimated that intraoral cavity biofilms consist of at least 800 types of bacteria. Therefore, it is suggested that this medicinal plant has a high potential for preventing dental disease and aspiration pneumonitis in geriatric patients and recovering patients when it is added to mouthwash and toothpaste [163].

The in vitro antibacterial potential of *D. blancoi* was also found against biofilm formation by *S. mutans*. Both extracts containing tannins and other phenols showed inhibition ranges of 96% for methanol and 95% for ethyl acetate [164].

Recently, *Diospyros* species rich in tannins have been applied in the development of nanoparticles. For instance, titanium dioxide (TiO_2_) nanoparticles containing *D. ebenum* leaf extract exhibit excellent antibacterial activity and potential against Gram-negative bacteria *E. coli* [165]. Silver nanoparticles (AgNPs) containing aqueous extract from the fruit of *D. malabarica* have demonstrated antibacterial activity against *S. aureus* at 500 μg/mL and against *E. coli* at 1000 ug/mL, with an average zone of inhibition size of 8.4 ± 0.3 mm and 12.1 ± 0.5 mm and 6.1 ± 0.7 mm and 13.1 ± 0.5 mm, respectively [166]. Similarly, biogenic silver nanoparticles demonstrated excellent antibacterial activity against a broad range of bacteria, with the highest antibacterial activity observed against *E. faecalis* (17.77 mm) and *B. subtilis* (20 mm), also demonstrating good hemocompatibility against humans and rat red blood cells [167].

##### Antiviral Activity

No studies were found on the specific activity of tannins isolated from the native *Diospyros* species in Mozambique. However, a tannin isolated from *D. kaki* has been demonstrated to have in vitro antiviral activity against the influenza virus, vesicular stomatitis virus, poliovirus, coxsackievirus, adenovirus, rotavirus, feline calicivirus, mouse norovirus, Sendai virus, and Newcastle disease virus [168]. The results of another study involving *D. kaki* extracts with tannin contents ranging from 0.08 to ≥0.11 mg/mL demonstrated their capacity to inactivate human noroviruses and bacteriophage MS2, both of which are the cause of gastroenteritis and foodborne illnesses worldwide (i.e., the results suggest that the antiviral effect and astringent effects of tannins are likely related to noroviral genome reduction and MS2 inactivation) [169].

##### Antiparasitic Activity

Species of the genus *Diospyros* contain a broad spectrum of antimicrobial agents identified using in vitro and/or in vivo methods against strains capable of causing opportunistic infections as well as neglected parasitic diseases. The anthelmintic activity of a *D. peregrina* fruit extract containing tannins was compared to the standard drug albendazole. The extract was found to be more potent than the selected standard drug at a concentration of 10 mg/mL [170].

According to the WHO, malaria is one of the most widespread neglected diseases in Africa, caused by the parasite *Plasmodium* and responsible for severe immune complications and deaths. The anti-*Plasmodium* activity of extracts from various species of the Mozambican *Diospyros* species has been reported in the literature. Ethyl acetate extract from *D. abyssinica* leaves showed moderate activity against chloroquine-resistant *Plasmodium falciparum* (FcB1), while *D. mespiliformis*, traditionally used to treat malaria, showed potent antimalarial activity in mice infected with *Plasmodium berghei* and significant inhibition of beta-hematin using an in vitro assay [98].

The antiparasitic activity against *Leishmania donovani*, *Trypanosoma cruzi*, and *Trypanosoma brucei* was confirmed in several studies on *Diospyros* species [99]. For example, an acetate leaf extract of *D. abyssinica* and the isodiospyrin and diospyrin marker compounds isolated from the bark by bioguided fractionation showed high anti-*L. donovani* activity (IC_50_ = 1.5 g/mL, extract, and IC_50_ = 0.5 g/mL, isolated compounds) [65].

## 3. Materials and Methods

This review was conducted according to the criteria described in the Preferred Reporting Items for Systematic Reviews and Meta-Analyses (PRISMA) 2020 statement (http://www.prisma-statement.org/; accessed on 16 January 2023). For this purpose, the scientific literature data were considered until 10 December 2022.

### 3.1. Search Strategy

The scientific data were collected using the search engines PubMed, Scopus, Web of Science, and Google Scholar, identifying all scientific papers published between 1 January 1970, and 10 December 2022 using the keywords Diospyros AND antibacterial, Diospyros AND antifungal, Diospyros AND antiparasitic, Diospyros AND antiviral, Diospyros AND medicine, Diospyros AND chemical compounds, Diospyros AND biological activity, and Diospyros AND toxicity.

### 3.2. Study Selection

**Figure 7:**
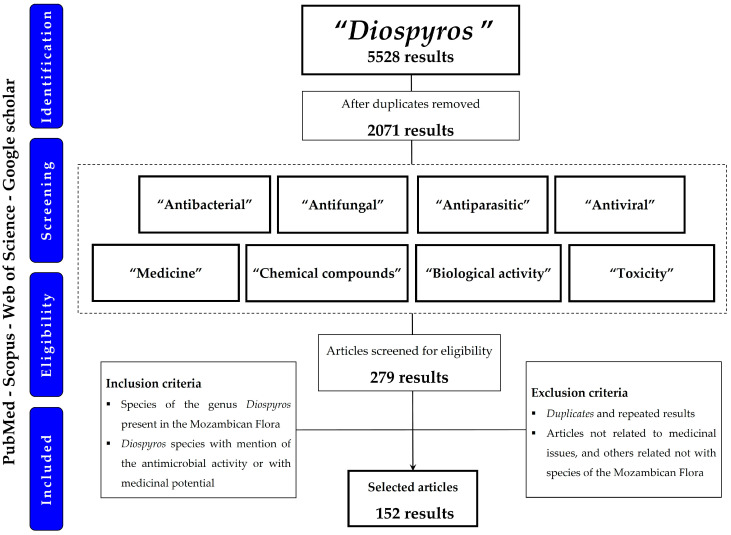
PRISMA flowchart of the screening process in the different databases.

As described in Figure 7, a total of 5528 scientific studies were included in the search and initial data collection based on their title and abstract. After eliminating the duplicates, 2071 studies remained, of which 1852 could not be selected due to a lack of information relevant to this work. After the screening, 279 studies reporting on *Diospyros* were considered eligible for inclusion in this review.

#### 3.2.1. Criteria for Inclusion and Exclusion of Data

##### Inclusion Criteria

▪ Related to the *Diospyros* genus, in particular species of the genus *Diospyros* present in Mozambican Flora;
▪ Abstract or full text in English;
▪ Studies on *Diospyros* species concerning their medicinal importance.

##### Exclusion Criteria

▪ Duplicate scientific publications;
▪ Not directly related to medicinal issues and others related but not with species of Mozambican Flora;
▪ Containing irrelevant or incomplete information.

## 4. Conclusions

Species of the genus *Diospyros* have been studied worldwide, with a significant number exhibiting pharmacological activity. One referenced example, *D. kaki*, native to East Asia, NaoXinQing, is part of a patented and officially approved traditional Chinese medicine formula for the treatment of stroke. However, there are no studies integrating data on all *Diospyros* species present in the flora of Mozambique.

More than 70% of Mozambique’s population uses medicinal plants for primary health care, and a total of 54.8% of the *Diospyros* species used in the country’s ethnomedicine are also used in other regions of Africa; however, the biological potential of most of them is still largely unknown. For example, 64.5% of these species were not tested for their antibacterial properties, namely *D. abyssinica* subsp. *attenuata*, *D. abyssinica* subsp. *Chapmaniorum*, *D. anitae*, *D. consolatae*, *D. consolatae-rotundifolia intermediates*, *D. dichrophylla*, *D. ferrea*, *D. inhacaensis*, *D. kirkii*, *D. kirkii-mespiliformis intermediates*, *D. loureiriana* subsp. *Loureiriana*, *D. natalensis* subsp. *Numulária*, *D. quiloensis*, *D. senensis*, *D. truncatifolia*, *D. usambarensis* subsp. *Usambarensis/rufescens*, *D. villosa* var. *parvifolia*, *D. villosa* var. *villosa*, *D. whyteana*, *D. zombensis*, and *Diospyros* sp. no. 1 sensu FZ. On the other hand, several isolated compounds of these species (particularly naphthoquinones and triterpenoids) have also been isolated from other species of the genus *Diospyros*, showing different biological activities including antiviral activity. However, no antiviral studies were found on the Mozambican species.

Studies on the antifungal potential of *Diospyros* are still scarce. In fact, the antifungal activity of 98.14% of the species (*D. abyssinica* subsp. *attenuata*, *D. abyssinica* subsp. *chapmaniorum*, *D. anitae*, *D. bussei*, *D. consolatae*, *D. consolatae-rotundifolia intermediates*, *D. dichrophylla*, *D. inhacaensis*, *D. kabuyeana*, *D. kirkii*, *D. kirkii-mespiliformis intermediates*, *D. loureiriana* subsp. *loureiriana*, *D. lycioides* Desf. subsp*. sericea*, *D. natalensis* subsp. *natalensis*, *D. natalensis* subsp. *numulária*, *D. quiloensis*, *D. rotundifolia*, *D. senensis*, *D. squarrosa*, *D. truncatifolia*, *D. verrucosa*, *D. villosa* var. *parvifolia*, *D. whyteana*, *D. zombensis*, and *Diospyros* sp. No. 1 sensu FZ) need to be evaluated, as they are traditionally used to treat skin diseases and diseases of the oral cavity, as well as other diseases where opportunistic fungal infections can co-occur. In addition, antiparasitic activities have been studied in other species of the genus *Diospyros*, however, 97.21% of Mozambican species (*D. abyssinica* subsp. *attenuata*, *D. abyssinica* subsp. c*hapmaniorum*, *D. anitae*, *D. consolatae*, *D. consolatae-rotundifolia intermediates*, *D. dichrophylla*, *D. ferrea*, *D. inhacaensis*, *D. kirkii*, *D. kirkii-mespiliformis intermediates*, *D. lycioides* Desf. subsp. *sericea*, *D. mafiensis*, *D. natalensis* subsp. *numularia*, *D. quiloensis*, *D. rotundifolia*, *D. senensis*, *D. squarrosa*, *D. truncatifolia*, *D. villosa* var. *villosa*, *D. villosa* var. *parvifolia*, *D. whyteana*, *D. zombensis*, and *Diospyros.* sp. no. 1 sensu FZ) have not yet had their antiparasitic activities studied.

In summary, out of the 31 native and naturalized species in the flora of Mozambique that are used in different regions of Africa, a total of 17 species have not been studied as antimicrobial agents, of which three species, namely *D. dichrophylla*, *D. whyteana*, and *D. zombensis*, have only been studied at the toxicological level. Of the 14 species that have already been the subject of antimicrobial studies, *D. abyssinica* and *D. mespiliformis* are the best studied.

This work provides comprehensive information on the chemical, biological, and toxicological studies of the *Diospyros* species present in the flora of Mozambique, examining their pharmacological potential in detail. Of the *Diospyros* plant parts, the root is the best-researched and documented. The identified studies confirmed ongoing efforts to improve the understanding of the mechanism of action underlying the biological activity, and in particular, the antimicrobial activity of these species, drawing on their traditional use. In addition, several secondary metabolites of *Diospyros* are currently being investigated for their potential pharmacological applications. However, it is important to emphasize that most of the available data are in vitro assessments of biological activity. Therefore, further efforts are needed to obtain more comprehensive evidence aimed at strengthening the validity and applicability of the results and ultimately contributing to public health benefits, especially in the face of global antimicrobial resistance.

## Figures and Tables

**Table 1:** Species of the genus *Diospyros* L. present in Mozambican Flora.

First Discription Year	Scientific Name	Author	Common Name English/Local	MD ^1^								IUCN ^2^
				N	Z	T	MS	GI	M	CD	Np	
1980	*D. anitae*	F.White	malawi star apple/-									LC
1911	*D. bussei*	Gürke	coral star-berry/-									NT
1935	*D. consolatae*	Chiov.	-/novolo									LC
1963	*D. dichrophylla*	(Gand.) De Winter	poison star-apple/-									LC
1933	*D. ferrea*	(Willd.) Bakh.	-/-									A
1962	*D. inhacaensis*	F.White	coastal jackal-berry/dodo									LC
1988	*D. kabuyeana*	F.White	-/-									LC
1873	*D. kirkii*	Hiern	large-leaved jackal-berry/ cula, fuma, jacualala, mucula, murriba, tendje									LC
1980	*D. mafiensis*	F.White	-/-									NT
1844	*D. mespiliformis*	Hochst. ex A.DC.	african ebony, jackal-berry/ muribariba, mucula, muquéué, murriparipa, mutona, mussuma					∇				LC
1956	*D. quiloensis*	(Hiern) F.White	crocodile-bark jackal-berry/ midodo, murodo									LC
1873	*D. rotundifolia*	Hiern	dune star-apple/ impapa, mapiti, munhentze				Δ					NE
1861	*D. senensis*	Klotzsch	spiny jackal-berry/ matamba, mudalima, tombatica									LC
1861	*D. squarrosa*	Klotzsch	rigid star-berry/cachenz’ere, mpomopo, senzasicana, sicana									LC
1980	*D. truncatifolia*	Caveney	square-leaved star apple/ impope, mpope									LC
1873	*D. verrucosa*	Hiern	warty star-apple/djacola, mkonhomo, nkalanongo, riparipa									LC
1961	*D. whyteana*	(Hiern) P.White	bladder-nut/-									LC
1963	*D. zombensis*	(B.L.Burtt) F.White	malawi star-apple/-									LC
1891	*D. abyssinica* subsp. *abyssinica*	(Hiern) F.White	giant diospyro*s*/-									LC
1988	*D. abyssinica* subsp. attenuata *	(Hiern) F.White	giant diospyros/-									LC
1980	*D. abyssinica* subsp. *chapmaniorum*	(Hiern) F.White	giant diospyros/-									LC
1837	*D. loureiriana* subsp. *loureiriana* ^a^	G.Don	dye star-apple, sand star-apple/chipongoti, nhandima									LC
1805	*D*. *lycioides* Desf. subsp. *sericea*	(Bernh.) De Winter	eastern blue-bush, red star-apple/chitomatomana, m’dima									LC
1968	*D. natalensis* subsp. *natalensis* *	(Harv.) Brenan	acorn jackal-berry/-									A
2009	*D. natalensis* subsp. *nummularia*	(Harv.) Brenan Jordaan	acorn diospyros, acorn jackal-berry, granite jackal-berry **/-									A
^b^ *D. usambarensis* subsp. *usambarensis /rufescens*		F.White	-/aboba, kidanko, mpome, nhamudima, popa									LC
^c^ *D. villosa* (L.) var. *villosa*		De Winter	hairy star-apple/nhachibabane, nhaurratane, chicanela, chicumbela, chibabane									A
^c^ *D. villosa* var. *parvifolia*		(De Winter) De Winter	hairy star-apple/-									A
^d^ *D. consolatae-rotundifolia intermediates*		-	-									A
^d^ *D. kirkii-mespiliformis intermediates*		*-*	-									A
^d^ *D.* sp. no. 1 sensu FZ		-	-									A

^1^ Distribution in Mozambique (blue, MD) [10,11]; Common name local (green, MD) [12]: N—Niassa; Z—Zambezia; T—Tete; MS—Manica and Sofala; GI—Gaza–Inhambane; M—Maputo; CD—Cabo Delgado; Np—Nampula; * Not identified; ** Other names—small-leaved jackal berry, Tickey tree; ∇: Gaza; Δ: Sofala. ^2^ International Union for the Conservation of Nature: LC—least concern; NE—not evaluated; NT—near threatened [B2ab(iii)]; A—absent. WFO Plant List: ^a^ *D. loureiroana* G.Don subsp. *loureiriana*; ^b^ Synonym of *D. loureiroana* subsp. *rufescens* (Caveney) Verdc.; ^c^ Synonym of *D. villosa* (L.) De Winter; ^d^ not included in the WFO Plant List [10,11]; (-)—not available.

## Data Availability

Not applicable.
